# Methyl quantum tunneling in ionic liquid [DMIm][TFSI] facilitated by Bis(trifluoromethane)sulfonimide lithium salt

**DOI:** 10.1038/s41598-018-28756-5

**Published:** 2018-07-09

**Authors:** Changwoo Do, Xiao-Guang Sun, Charl J. Jafta, Sheng Dai, Michael Ohl, Eugene Mamontov

**Affiliations:** 1 Neutron Scattering Division, Oak Ridge National Laboratory, Oak Ridge, TN 37831 USA; 2 Chemical Science Division, Oak Ridge National Laboratory, Oak Ridge, TN 37831 USA; 3 Forschungszentrum Jülich, 52428 Jülich, Germany

## Abstract

We probe, for the first time, quantum tunneling in the methyl groups of the ionic liquid [DMIm][TFSI] facilitated by the presence of Bis(trifluoromethane)sulfonimide lithium salt. The observation of tunneling is made possible by crystallization, rather than vitrification, of [DMIm][TFSI] at low temperature. Neutron scattering measurements detect quantum tunneling excitations at ~27 μeV at temperatures below 30 K in the presence of LiTFSI at a concentration of 1 mol/kg, but not in salt-free [DMIm][TFSI]. This indicates that the methyl rotational potential barrier is reduced by the presence of LiTFSI, thus bringing the tunneling excitations into the measurable range. The salt-induced reduction of the rotational barrier is corroborated by quasi-elastic scattering data associated with stochastic re-orientation of methyl groups measured between 40 and 60 K.

## Introduction

Ionic liquids (ILs) have been attracting tremendous interest as solvents with many interesting qualities. In particular, their good thermal and chemical stability, low melting point, low flammability, and high ionic conductivity are ideal for replacing existing electrolyte media in lithium batteries. For example, ILs with bis(tri-fluoromethanesulfonyl)imide (TFSI) anions are popularly used for their good stability and conductivity, as well as the ability to form solid electrolyte interphases (SEIs)^1–4^.

ILs are also popularly used to study glass transition and ionic conductivity relaxation due to the wide range of chemical and physical properties available from different choices of cations and anions. In these research areas, dynamics of both structural and interionic correlation have been studied mainly by neutron scattering^5,6^, or dielectric spectroscopy^7,8^. In addition, plastic crystals formed by ILs at low temperature are known to exhibit a unique ion conduction mode through coupling to rotation dynamics (especially methyl groups)^9^. Therefore, understanding these molecular dynamic properties is expected to provide critical knowledge to reveal the details of the microscopic interactions in the ILs, as well as ion transport mechanisms of solid electrolytes based on plastic crystals.

**Figure 1:**
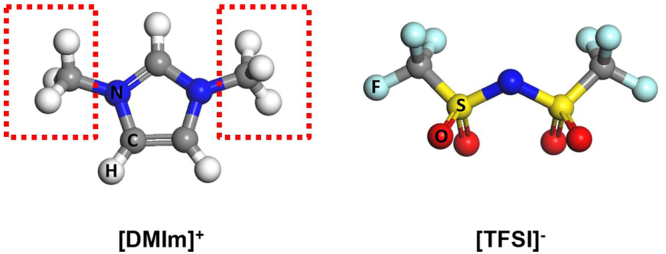
Molecular structure of [DMIm]^+^ and [TFSI]^−^. Methyl groups that exist in [DMIm]^+^ are indicated with dotted rectangles.

Here, we found that the methyl groups, which are often present in many ILs, can be used as a probe to obtain information about characteristics of the surrounding molecular arrangement. In particular, we investigated the behavior of methyl groups in 1,3 dimethylimidazolium bis(tri-fluoromethanesulfonyl)imide ([DMIm][TFSI]) (Fig. 1). This was made possible through observation of the quantum tunneling peaks in the studied IL system. The rotation dynamics of molecular rotors, such as methyl groups, have been a popular subject of study for neutron scattering experiment^10–12^. Since the rotational potential is described by the energy landscape of the surrounding molecules, measurement of the quantum tunneling frequency can provide details about the characteristics of the local environment. In ILs, however, quantum tunneling has never been measured, nor reported so far, largely due to the typically glassy behavior of the ILs that often precludes crystallization on cooling down. Because the rotational potential barriers of methyl groups can vary due to the local disorder, glassy state ILs would exhibit too broad distribution of tunneling frequencies. We found that [DMim][TFSI] crystalizes at low temperature, which makes it possible for the quantum tunneling peaks to be observed.

## Results and Discussion

**Figure 2:**
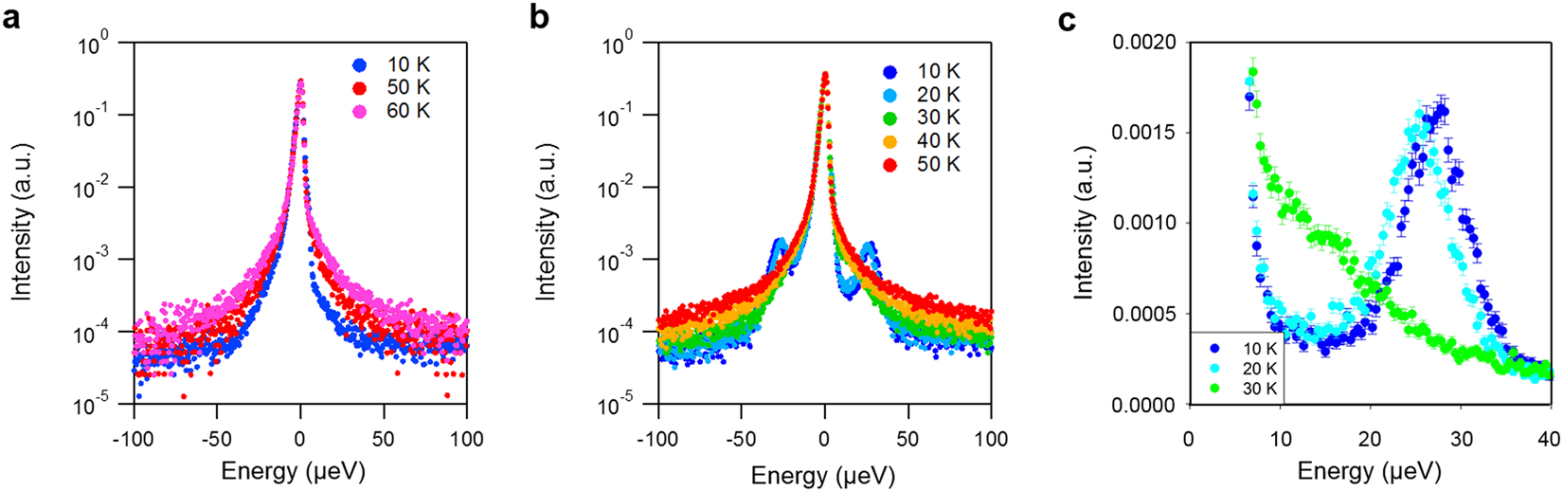
QENS spectra of [DMIm][TFSI] without (**a**) and with (**b**) additional LiTFSI. (**c**) Closer look of the QENS spectra around the tunneling peaks.

The location of methyl groups in [DMim][TFSI] is indicated in Fig. 1, where the molecular structures of DMIm and TFSI are described. Figure 2 shows the QENS spectra of samples at various temperatures. The spectra sharpen as the temperature is decreased, indicating suppression of motions in the system. Interestingly, on top of the QENS signal, sharp inelastic peaks are observed around 27 µeV from the IL with LiTFSI at temperatures below 30 K. The peaks visible in Fig. 2b exhibit characteristic properties of quantum tunnelling excitations, such as downward shift and eventual disappearance into the quasi-elastic signal as the temperature is increased.

These trends in the temperature dependence of the inelastic excitations can be better visualized from Fig. 2c that uses linear scale and truncated elastic peak. It is evident that, as the temperature is increased, the inelastic peak does not increase in intensity, as would be required for vibrational motions obeying Bose statistics. Instead, the observed temperature behavior is characteristic of systems exhibiting quantum tunneling, where the peak intensity decreases with temperature due to transition from the lowest to higher split level of the ground (as opposed to vibrationally excited) state^13^.

**Figure 3:**
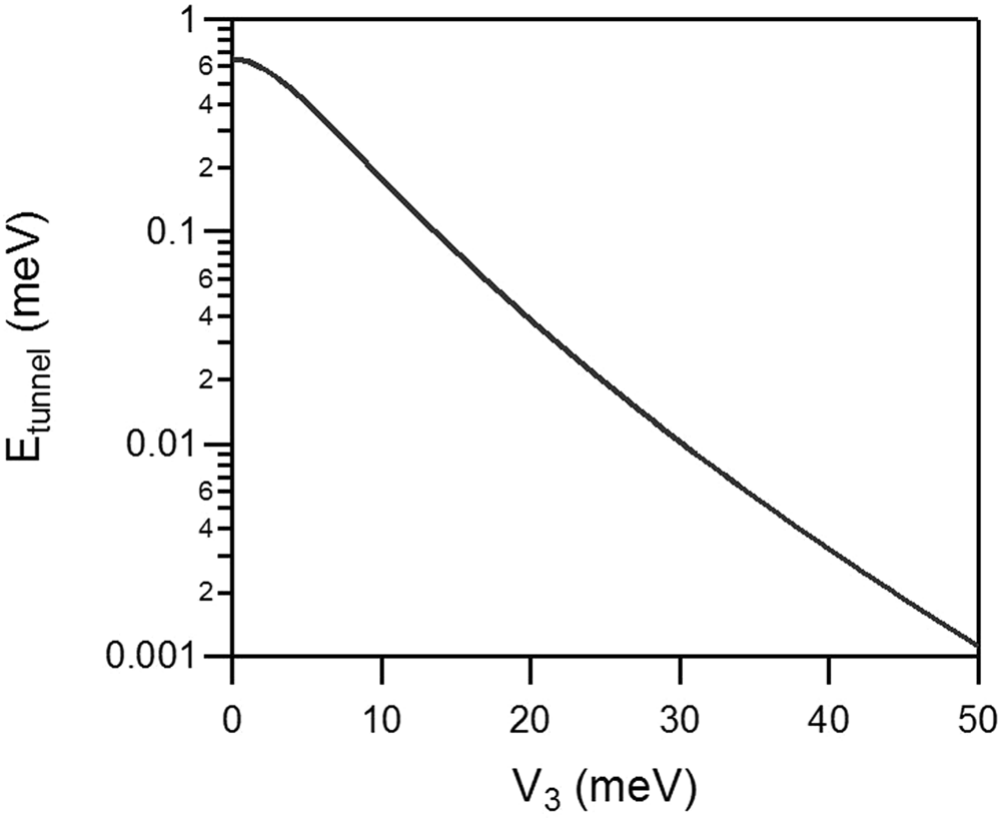
Tunnel splitting energy calculated as a function of potential barrier (V_3_).

**Figure 4:**
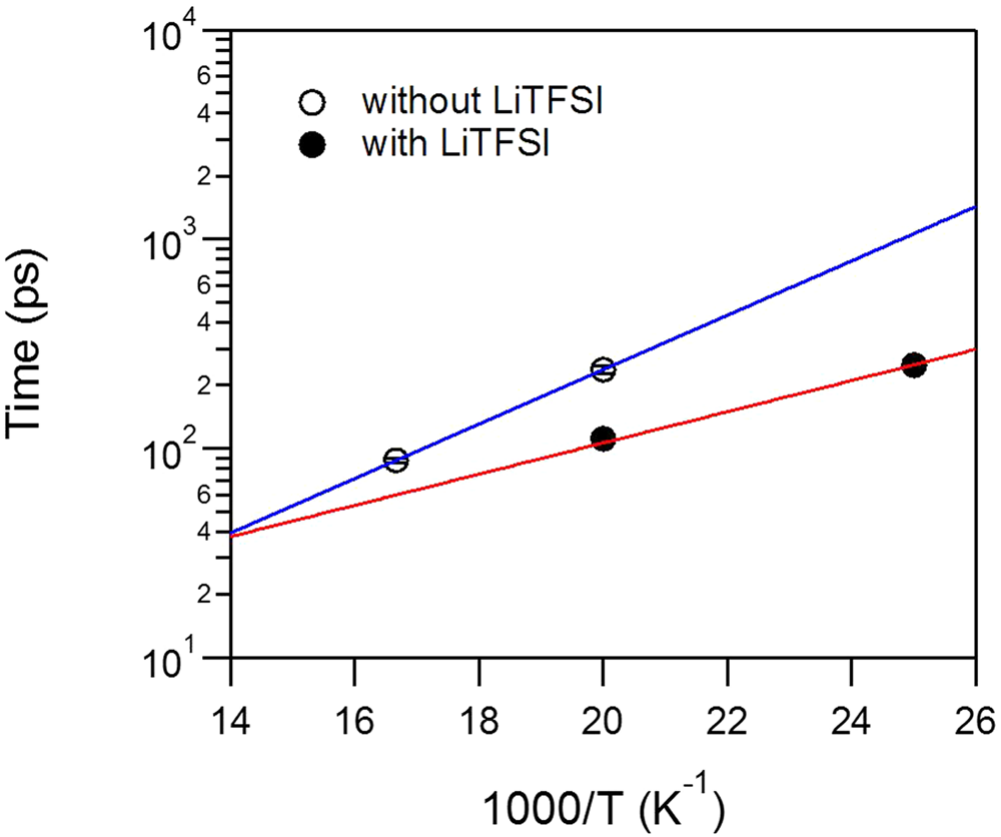
Symbols are activation energy plots for methyl group rotation jump times obtained from QENS experiment. Lines are fits with Arrhenius model.

Considering that the quantum tunnelling frequencies have exponential dependence on the barrier height, a decrease in activation energy introduced by the addition of Li+ ions must have lowered the barrier for the 3-fold methyl jumps enough so that the tunnelling energy becomes visible within the energy window of the experiment. The quantum tunnelling peaks of the methyl groups in the salt-free IL must be present, but buried within the resolution line of the spectrometer and thus undetectable. From the position of the tunnelling peaks (~27 µeV at 20 K), the barrier height of 23 meV can be estimated as follows. The quantum tunnel splitting energy is calculated by solving energy eigenvalues of the total Hamiltonian of methyl rotor^14–16^:

1 $${{\rm{H}}}_{{\rm{mn}}}=\frac{{\hslash }^{2}{n}^{2}}{2I}{\delta }_{mn}+\frac{{V}_{3}}{4}\{2{\delta }_{n,m}-{\delta }_{n-m+3,0}-{\delta }_{n-m-3,0}\}$$

where $I$ is the moment of inertia of CH_3_ and ${V}_{3}$ is the potential barrier for rotation. Here, ${\hslash }^{2}/2I\,\,$is known as 0.654 meV and n,m = 0, ±1, ±2, … From the curve presented in Fig. 3, the potential barrier of ~23 meV corresponds to the 27 µeV tunneling peaks observed from the experiment data (Fig. 2b). Likewise, the barrier height in the salt-free IL must be higher than ~40 meV for the tunneling peaks to be buried within the resolution line of FWHM of 3.5 µeV. The activation energy for methyl group rotation can be probed independently, using the rotation jump times (inverse width of the measured QENS signal) as shown in Fig. 4. Indeed, this activation energy decreases by almost a factor of 2 by adding LiTFSI, from 25.8 meV to 14.8 meV.

**Figure 5:**
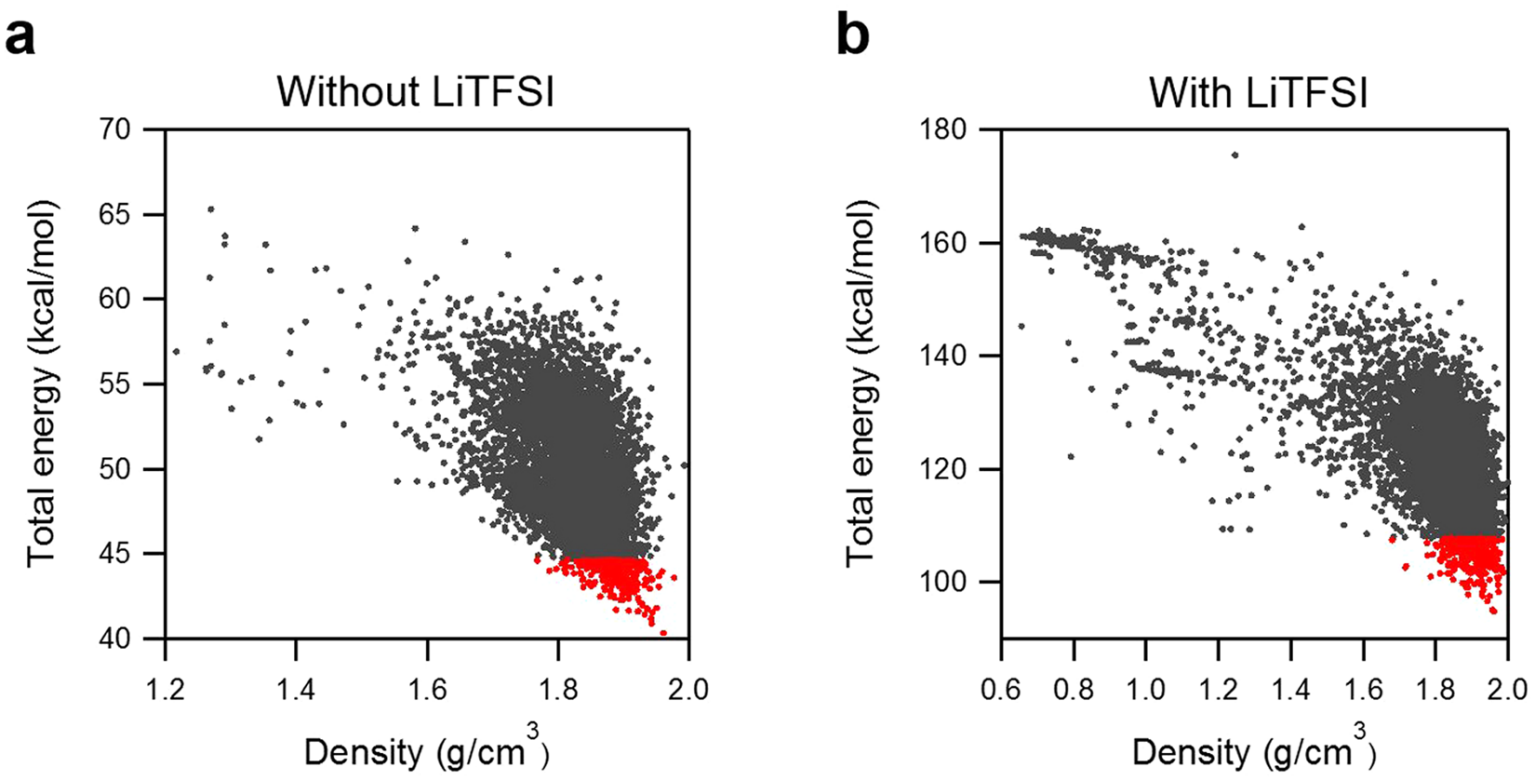
Total energy and density of each configuration predicted for P21/c space group. Total 6432 and 5992 configurations were predicted for without (**a**) and with (**b**) LiTFSI systems, respectively. Red markers indicate 300 lowest energy configurations. Here, the total energy is per asymmetric unit of the system.

To our knowledge, this is the first time that quantum tunnelling has been observed in ILs. One factor that made this observation possible was crystallization, rather than typically observed vitrification on cooling down^6^, of [DMIm][TFSI], with or without salt. Besides, the presence of salt lowered the energy barrier of methyl group rotations, thereby bringing the quantum tunnelling excitations within the range of the experiment. To further understand how LiTFSI changes molecular environment around methyl groups, especially in general case, when vitrification leads to a variety of local environments that precludes experimental observation of quantum tunnelling, we have employed computer simulation module called Polymorh using Materials Studio 2016 (BIOVIA, USA) with the COMPASS force field to predict potential crystallization structures^17,18^. Crystal structure prediction using Polymorph module in Materials Studio utilizes Monte-Carlo based configuration search to find structures that can minimize Gibbs free energy. To be more specific, the entropic contribution is in fact neglected as an approximation for practical calculation. Therefore, contribution to the energy comes from the intramolecular energy, intermolecular electrostatic energy, and repulsion-dispersion interaction energy, which depend on the conformation of the molecules and their relative positions within the unit cell. Here, we limited our search to selected space groups including P2_1_/c, P1, P2_1_, C2/c, C2, and Cc. Each space group predicted from a few hundred to several thousand of potential crystal structures. As an example, the total energy and the density of the predicted crystal structures for P2_1_/c space group is shown in Fig. 5. For pure [DMIm][TFSI], a total of 6432 configurations were predicted, with total energy ranging from 40 kcal/mol to 65 kcal/mol. Overall density of low-energy configurations was highly concentrated around 1.8 g/cm^3^ to 1.9 g/cm^3^ range. With additional LiTFSI, 5992 configurations were searched. Total energies were found to be in the range from 90 kcal/mol to 160 kcal/mol. Low-energy configurations exhibit slightly higher density distribution, between 1.8 g/cm^3^ and 2.0 g/cm^3^, compared to the pure IL. In both predictions, crystal configurations with much lower density were predicted when the total energy is high, indicating they are far from realistic structural configurations. However, when comparing the total energy of predicted crystal structures with and without LiTFSI, special attention needs to be paid, since the total energy in Fig. 5 is in kcal/mol per asymmetry unit. For the [DMIm][TFSI] with LiTFSI, there are more molecular units in the unit cell. Therefore, the cell volume is overall larger than that of the pure IL. Later, when the potential barrier is compared, we will use total energy per a methyl group instead of the units used in Fig. 5. This will provide more reasonable basis for comparing total energy changes.

**Figure 6:**
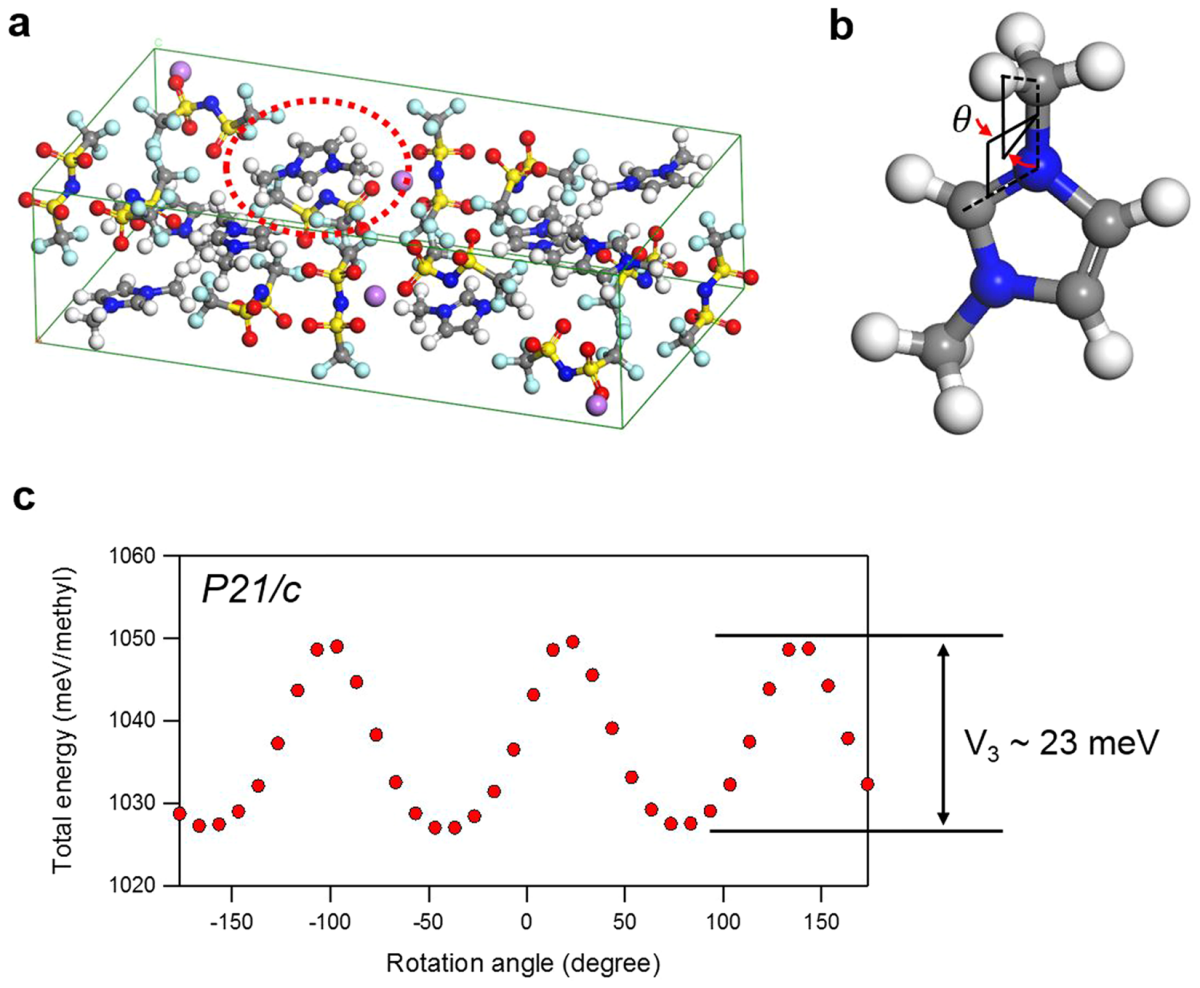
(**a**) An example of the predicted crystal structure of DT-1 which belongs to P2_1_/c space group. (**b**) Rotation angle of the methyl group is described. (**c**) Total energy per methyl group as a function of methyl group rotation. The potential barrier is estimated to be around 23 meV in this example.

The potential barrier as a function of the methyl group rotation was calculated by approximating the methyl group as a rigid rotor. As an example, one of the methyl groups is depicted for the lowest energy configuration of P21/c space group predicted for [DMIm][TFSI] with LiTFSI (Fig. 6a). The total energy is calculated by varying the torsion angle shown in Fig. 6b from negative 180 to positive 180 degrees. In doing this, we considered the methyl groups bonds rigid, and no further relaxation was performed. The bond vibrations are not considered because the temperature dependency of the peak intensities observed in Fig. 2c excludes the possibility of vibrational origin. The total energy per methyl group as a function of rotation angle is shown in Fig. 6c. It demonstrates the three-fold symmetry of the potential barrier for the methyl group very well.

As we have mentioned above, the quantum tunnel splitting energy can be calculated by solving energy eigenvalues of the total Hamiltonian of methyl rotor^14–16^ (see Fig. 3). One of the predicted structures shown in Fig. 6 exhibits 23 meV potential barrier height. According to the estimation, the tunnel-splitting energy is expected to be 25.7 µeV with 23 meV potential barrier (Fig. 3). This is in agreement with the energy observed from experimental data, Fig. 2b. It should be noted that this tunnel splitting energy varies depending on the choices of configurations found from the polymorph prediction.

**Figure 7:**
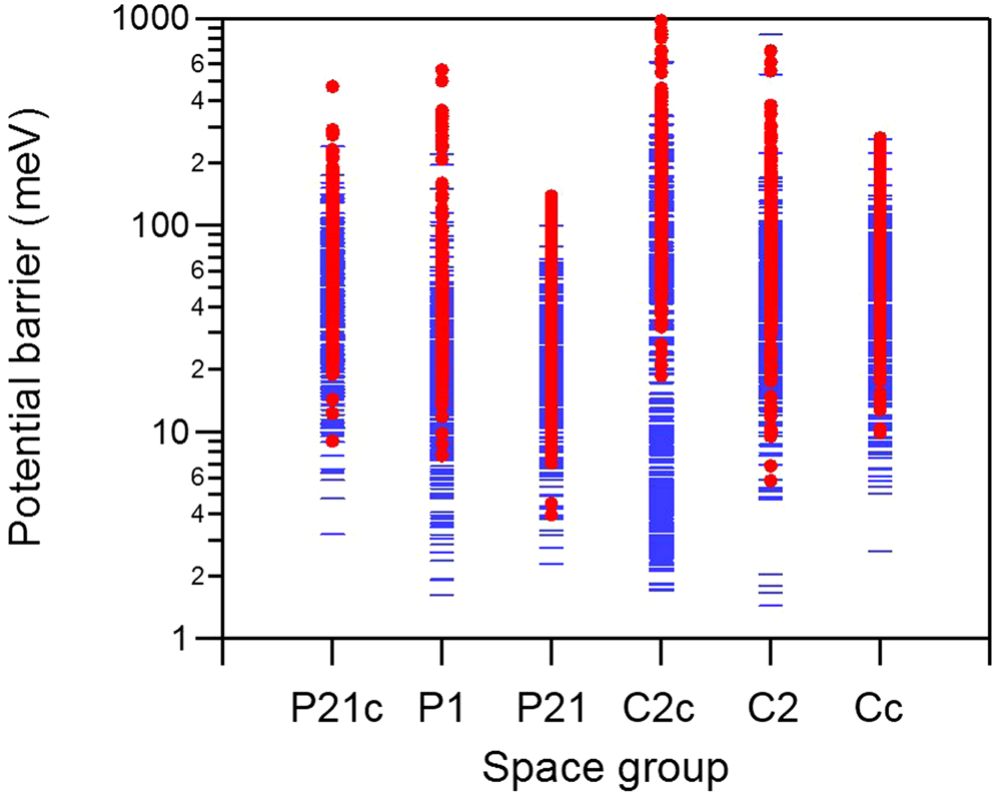
Potential barrier (${V}_{3}$) obtained from the predicted crystal structures are shown for with (blue minus) and without LiTFSI (red solid circle) systems. Only 300 lowest energy configurations are shown for each space group.

According to experimental data (Fig. 2), tunnelling peaks were only observed from ILs with LiTFSI. In order to understand what may be causing the appearance of tunnelling peaks in the presence of LiTFSI, we attempted to compare simulation results in more detail. However, as noted earlier, more than thousands of configurations are predicted as candidates for the crystal structure for individual space group (Fig. 5). Therefore, comparison between simulation results with LiTFSI and without LiTFSI has been made by i) estimating total energy as a function of rotation angle as in Fig. 6, ii) obtaining potential barrier (${V}_{3}$) for each configuration, iii) repeating (i) and (ii) for 300 lowest energy configurations for the selected space groups, and iv) plotting all the potential values in one plot for comparison. Figure 7 summarizes the results. Although the distribution is wide, it is evident that ILs with LiTFSI exhibit, overall, lower potential barrier for the methyl group rotation. Because the instrument resolution of QENS used in this experiment is 3.5 µeV, tunnelling energy lower than this value cannot be observed. In other words, tunnelling peaks in S(q, E) cannot be observed when the potential barrier is greater than 40 meV, which is likely the case for pure [DMIm][TFSI] system (red solid circles in Fig. 7).

**Figure 8:**
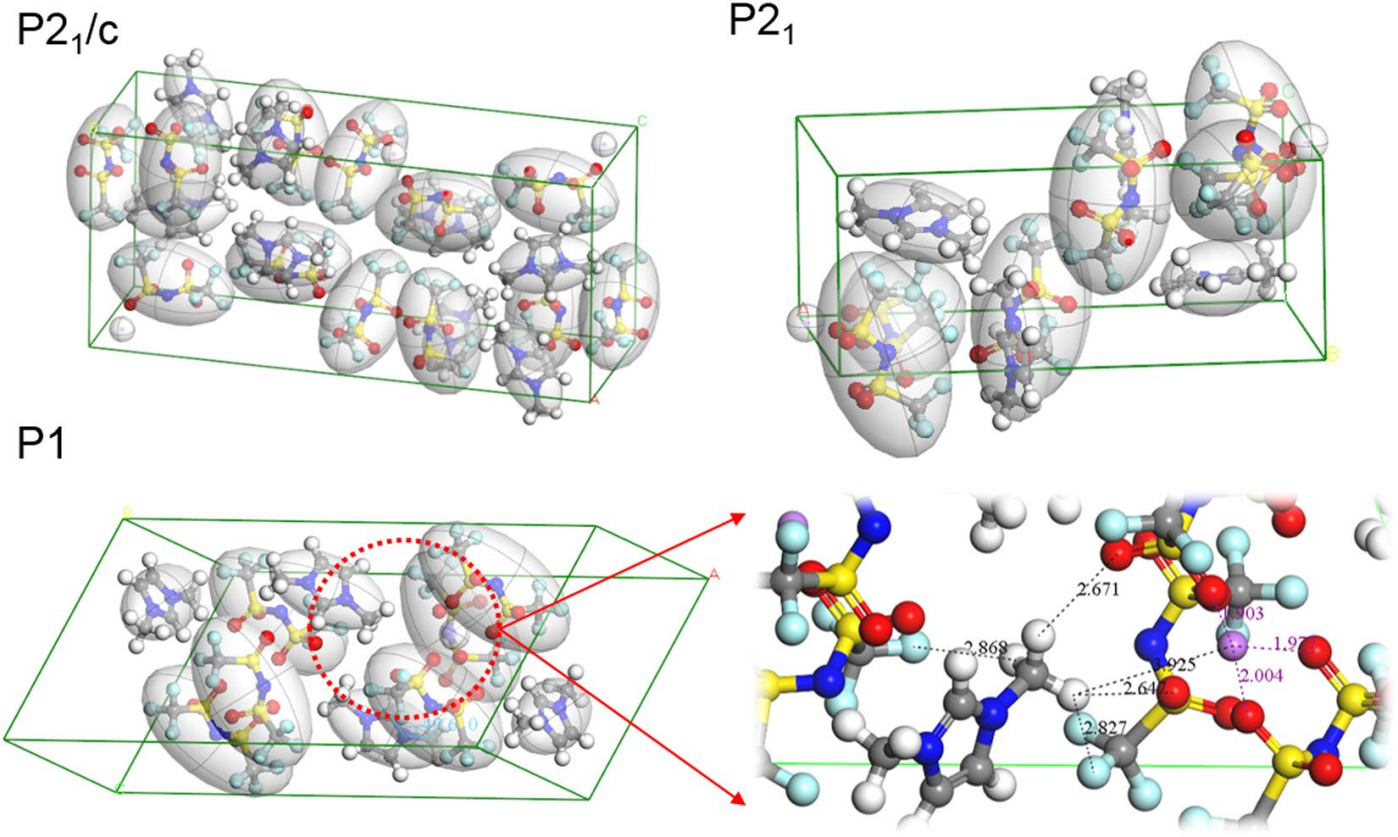
Lowest energy configurations for a few selected space groups. Visual inspection on these configurations confirms that methyl groups are under the influence of the TFSI- ions rather than Li+ ions directly. Examples of distances to the nearest atoms from the methyl group are shown for one of the configurations of P1 space group.

Our simulation results demonstrate the general effect of reduction of the potential barrier by the salt, which is highly relevant to the more typical case when crystallization in ILs does not take place on cooling down, thus precluding experimental observation of quantum tunneling effect. Furthermore, by examining the selected crystal structures whose potential barriers are around 25–30 meV, we noted that TFSI- ions are more closely located to the methyl groups of ILs than Li+ ions (Fig. 8). For example, in the lowest energy configuration of P1 space group shown in Fig. 8, the nearest neighbor atoms around the methyl groups are either oxygen or fluorine atoms of the TFSI- ions. Li+ ions are closely located with oxygen atoms of TFSI- due to electrostatic attraction. This suggests that the tunneling energy was brought in the measurable range via arrangement of TFSI- ions. The influence of the salt on the potential landscape experienced by methyl groups may be similar in other ILs, despite the fact that most of them do not crystallize, but undergo vitrification instead, which would preclude the quantum tunneling measurements as a direct probe.

In conclusion, we have presented a direct evidence of quantum tunneling of methyl groups in IL for the first time using neutron scattering experiment. Whether measured alone, or in the presence of additional lithium salt, LiTFSI, the [DMim][TFSI] crystalizes at low temperature, which makes possible observation of well-defined quantum tunneling peaks. Furthermore, Monte-Carlo based simulation for possible crystal structures has been employed to search for suitable molecular configurations in [DMim][TFSI] with and without LiTFSI. Examination over thousands of molecular configurations obtained from simulation suggests that the rotational potential is generally lowered by the addition of LiTFSI. Visual inspection of selected crystal structures indicates, rather unexpectedly, that TFSI- ions may play a greater role than Li+ ions in this effect. The origin of the observed tunneling effect may be identified via precise structural characterization in the future studies.

## Materials and Methods

1-methylimidazole and methyl iodide were purchased from Aldrich. [DMIm][TFSI] was synthesized and purified according to the reported procedure^19–21^. LiTFSI was purchased from Aldrich and dried at 150 °C under high vacuum for 12 hours before use. Calculated amount of LiTFSI was added to [DMIm][TFSI] to make the desired salt concentration inside the glovebox. For the [DMIm][TFSI] with LiTFSI, the molar ratio of each component was DMIm:TFSI:Li = 2.65:3.65:1. Quasi-elastic neutron scattering (QENS) measurements were performed at backscattering spectrometer BASIS^22^ of the Spallation Neutron Source facility (Oak Ridge National Laboratory) using a standard setup with an energy resolution of 3.5 µeV (full width at half maximum) and accessible range of energy transfers of ± 100 µeV. The data reported here were averaged over a range of scattering momentum transfer of 0.2 Å^−1^ < Q 1.2 Å^−1^.

The methyl rotation jump times were calculated as τ = (h/2π)/Γ, where Γ is half-width at half-maximum of the Lorentzian component of the quasielastic data fits with the following expression:

2 $$S(E)=[x(E)+\frac{(1-x){\rm{\Gamma }}}{\pi ({{\rm{\Gamma }}}^{2}+{E}^{2})}]+(AE+B)$$

convolved with the resolution function, R(E), measured from a vanadium standard. The first term in the square brackets represents the elastic line, and the (AE + B) term is a fit linear background. Quasielastic data fits were performed for the 50 and 60 K data for the IL without LiTFSI and 40 and 50 K data for the IL with LiTFSI, to ensure that the quasielastic broadening remains within the ±100 µeV energy range of the spectrometer and, at the same time, the spectra are not affected by the tunneling peak visible at lower temperatures.

### Data availability

The datasets analyzed during the current study are available from the corresponding author on reasonable request.

## References

[1] Giffin GA (2016). Ionic liquid-based electrolytes for ‘beyond lithium’ battery technologies. J. Mater. Chem. A.

[2] Shao Y (2013). Making Li-Air Batteries Rechargeable: Material Challenges. Adv. Funct. Mater..

[3] Di Lecce D, Brutti S, Panero S, Hassoun J (2015). A new Sn-C/LiFe0.1Co0.9PO4 full lithium-ion cell with ionic liquid-based electrolyte. Mater. Lett..

[4] Wu F (2015). Ionic liquid electrolytes with protective lithium difluoro(oxalate)borate for high voltage lithium-ion batteries. Nano Energy.

[5] Kofu M (2013). Heterogeneous Slow Dynamics of Imidazolium-Based Ionic Liquids Studied by Neutron Spin Echo. J. Phys. Chem. B.

[6] Mamontov E, Luo H, Dai S (2009). Proton Dynamics in *N*, *N*, *N* ′, *N* ′-Tetramethylguanidinium Bis(perfluoroethylsulfonyl)imide Protic Ionic Liquid Probed by Quasielastic Neutron Scattering. J. Phys. Chem. B.

[7] Jarosz G (2011). Glass Transition Dynamics of Room-Temperature Ionic Liquid 1-Methyl-3-trimethylsilylmethylimidazolium Tetrafluoroborate. J. Phys. Chem. B.

[8] Wojnarowska Z (2013). Decoupling of conductivity relaxation from structural relaxation in protic ionic liquids and general properties. Phys. Chem. Chem. Phys..

[9] Macfarlane DR, Huang J, Forsyth M (1999). Lithium-doped plastic crystal electrolytes exhibiting fast ion conduction for secondary batteries. Nature.

[10] Colmenero J, Moreno AJ, Alegría A (2005). Neutron scattering investigations on methyl group dynamics in polymers. Prog. Polym. Sci..

[11] Moreno AJ (2001). Methyl group dynamics in glassy toluene: A neutron scattering study. J. Chem. Phys..

[12] Parker SF (2015). Methyl tunnelling of adsorbed methoxy on alumina catalysts. Chem. Commun..

[13] Kolesnikov AI (2016). Quantum Tunneling of Water in Beryl: A New State of the Water Molecule. Phys. Rev. Lett..

[14] Dimeo RM (2003). Visualization and measurement of quantum rotational dynamics. Am. J. Phys..

[15] Horsewill AJ (1992). Rotational tunnelling in organic molecules. Spectrochim. Acta Part A Mol. Spectrosc..

[16] Prager M, Heidemann A (1997). Rotational Tunneling and Neutron. Spectroscopy: A Compilation.

[17] Vasileiadis M, Pantelides CC, Adjiman CS (2015). Prediction of the crystal structures of axitinib, a polymorphic pharmaceutical molecule. Chem. Eng. Sci..

[18] Akkermans RLC, Spenley NA, Robertson SH (2013). Monte Carlo methods in Materials Studio. Mol. Simul..

[19] Sun X-G (2013). Bicyclic imidazolium ionic liquids as potential electrolytes for rechargeable lithium ion batteries. J. Power Sources.

[20] Tokuda H, Hayamizu K, Ishii K, Susan MABH, Watanabe M (2005). Physicochemical properties and structures of room temperature ionic liquids. 2. variation of alkyl chain length in imidazolium cation. J. Phys. Chem. B.

[21] Liao C (2011). Physicochemical properties of imidazolium-derived ionic liquids with different C-2 substitutions. Phys. Chem. Chem. Phys..

[22] Mamontov E, Herwig KW (2011). A time-of-flight backscattering spectrometer at the Spallation Neutron Source, BASIS. Rev. Sci. Instrum..

